# Identification and Characterization of a *Bursaphelenchus xylophilus* (Aphelenchida: Aphelenchoididae) Thermotolerance-Related Gene: *Bx-HSP90*

**DOI:** 10.3390/ijms13078819

**Published:** 2012-07-16

**Authors:** Feng Wang, Zhiying Wang, Danlei Li, Qiaoli Chen

**Affiliations:** 1 College of Forestry, Northeast Forestry University, Harbin 150040, China; E-Mails: bursaphelenchus@gmail.com (F.W.); zyw0451@sohu.com (Z.W.); ttwgy_cql@163.com (Q.C.); 2 College of Natural Resources and Environment, South China Agricultural University, Guangzhou 510640, China

**Keywords:** *Bursaphelenchus xylophilus*, HSP90, heat shock, pine wilt, suppression subtractive hybridization

## Abstract

Temperatures directly influence the distribution and intensity of pine wilt disease caused by the pine wood nematode, *Bursaphelenchus xylophilus*. To date, however, little is known about the causation and mechanism of this influence. The molecular chaperone HSP90 is a key component that contributes to survival in the abiotic stress response. In this study, we investigated the relationship between the survival of *B. xylophilus* and the functionality of the HSP90 gene. *Bx-HSP90* was cloned from a suppression subtractive hybridization library. *In situ* mRNA hybridization showed that *Bx-HSP90* was constitutively expressed in response to all of the temperatures tested, and RT-PCR indicated that all of the temperatures could induce *Bx-HSP90* transcription, with the highest transcript level detected at 30 °C. The suppression of the *Bx-HSP90* transcript by RNA interference led to a 25% reduction in the number of nematodes at 30 °C after 44 h. Sharp declines in the survival of the RNAi-treated nematodes were observed after 8 days at 25 °C, 48 h at 30 °C and 24 h at 35 °C. Both heat shock and the knockdown of *Bx-HSP90* hindered the growth of the *B. xylophilus* populations. The results indicate that *Bx-HSP90* is essential for the survival of *B. xylophilus*, confirming the thermoregulatory function of the gene, and delineate the timeframe and temperature range within which the gene function occurs.

## 1. Introduction

*Bursaphelenchus xylophilus*, the causative agent of pine wilt disease, is considered to be native to North America and to have been introduced into Japan in the early 1900s [1,2]. This disease has devastated pine forests in eastern Asia for at least the last four decades [3]. Although the origin of the *B. xylophilus* introductions remains unclear, this pest was discovered in China in 1982, in Korea in 1988 [3,4] and most recently in Portugal [5].

Thermoregulation describes the adaptive responses of an organism to changes in the environmental temperature. For many parasites that are transmitted by a vector to their mammalian host or that have a free-living stage in the environment, the ability to adapt to a variety of ecological niches is an important determinant of their success [6]. For *B. xylophilus*, however, environmental factors may directly influence the distribution and intensity of pine wilt in North America [2], as these factors apparently influence native pine species in Japan. For example, temperatures higher than 28 °C inhibit *B. xylophilus* reproduction, whereas the nematode cannot reproduce above 33 °C [7].

The pervasive effects of temperature on biochemical and physiological processes are thought to play a fundamental role in shaping the distribution and abundance of organisms. Many genes have been found to be potential targets of adaptive evolution due to temperature. In particular, the heat shock proteins (HSPs) are the genes best characterized for their role in cell survival during periods of stress, times when their ability to bind to denatured or misfolded proteins is essential for the survival of the organism. Heat shock proteins also function during normal growth and division as chaperones for protein folding and transport. Among the HSPs, members of the heat shock protein 90 (HSP90) family are the most abundant and highly conserved. HSP90 has been shown to chaperone denatured proteins under stressful conditions and also proteins involved in signal transduction pathways, such as steroid receptors and protein kinases. The rapid evolution of the HSP90 gene correlates with many key cellular functions [8]. Additionally, HSP90 operates in vesicle transport and telomerase regulation [9], and HSP90 stress potentiates rapid cellular adaptation through the induction of aneuploidy [10].

In this study, we investigated the high-temperature tolerance of *B. xylophilus* and cloned its thermotolerance-related gene, *Bx-HSP90*. Because *HSP90* is known to function in thermotolerance, we concentrated on the role of the *Bx-HSP90* gene in the resistance to environmental high-temperature stress. Because temperatures above 28 °C affect *B. xylophilus* development [11], and its embryonic development is disrupted at 35 °C [3], we selected temperatures of 25, 30 and 35 °C to test the thermoregulatory function of the *Bx-HSP90* gene. We also compared the survivability and population growth between *Bx-HSP90*-suppressed and normal nematodes exposed to different high-temperature stress environments. We were particularly interested in determining whether *HSP90* functions in the survival strategy of *B. xylophilus* in response to environments of high-temperature stress.

## 2. Results and Discussion

### 2.1. cDNA Cloning and Homology Analysis

An 893-bp thermotolerance-related cDNA, homologous to *HSP90* and named *B40*, was found using subtracted cDNA libraries and the BLAST method. The sequence of *B40* and those of the products of a spliced leader (SL) primer PCR and a 3′-RACE provided the information necessary to clone a 1206-bp cDNA sequence of the HSP90 gene. The sequence was named *Bx-HSP90* and submitted to National Center for Biotechnology Information (NCBI) (accession No. **EF490991**). *Bx-HSP90* contains an open reading frame of 1089 bp, with a 10-bp 5′ untranslated region (UTR) and a 109-bp 3′ UTR featuring a likely polyA-addition signal. Overall, this HSP90 gene shows the greatest similarity to that of *Heterodera glycines* (**AAO14563**) (E Value: 1 × 10^−147^). The encoded protein of 362 amino acids is not predicted to contain a signal peptide, and a PSORTII analysis predicts a cytoplasmic location.

**Figure 1:**
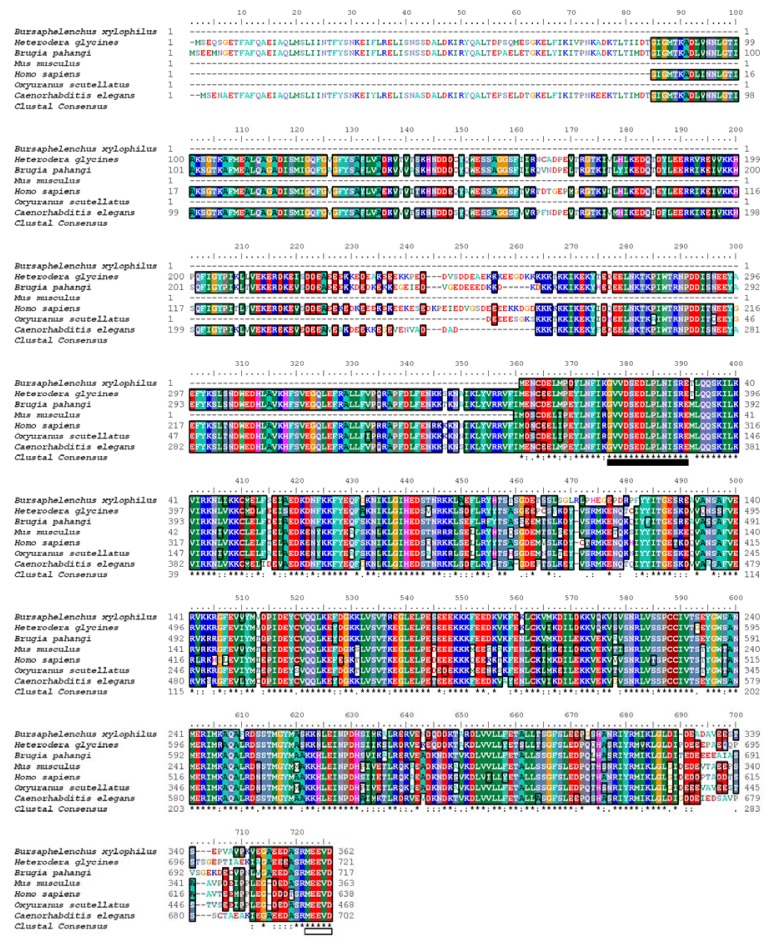
Alignment of *Bursaphelenchus xylophilus* HSP90 with homologs identified from the National Center for Biotechnology Information (NCBI) database. The alignment compares Bx-HSP90 with the HSP90 proteins of the soybean cyst nematode, *Heterodera glycines*, (AAO14563); the filarial nematode, *Brugia pahangi*, (CAA06694); the house mouse, *Mus musculus*, (AAH49951); the human, *Homo sapiens*, (AAH23006); the snake, *Oxyuranus scutellatus*, (AAY67995) and the free-living nematode, *Caenorhabditis elegans*, (NP_506626). NP_606626 is also known as an abnormal dauer formation family member. The accession numbers are those of the GenBank database. Identical amino acids are boxed. The asterisks, double dots and single dots denote the fully conserved, strongly conserved and weakly conserved amino acid residues, respectively.

An alignment of Bx-HSP90 with its closest homologs is shown in Figure 1. The molecular architecture and sequence of the Bx-HSP90 protein are similar to those of its closest homologs; the similarity occurs particularly in the C-terminal portion of the protein and is concentrated on the active site residues. A pairwise comparison of the predicted amino acid sequence of Bx-HSP90 with the sequence of each of the other HSP90s revealed a high degree of conservation along the entire length of the HSP90 sequences. The respective percentages of the compared sequences that were identical and similar were 79 and 89 for *H. glycines*, 80 and 89 for *Brugia pahangi*, 74 and 86 for *Mus musculus*, 72 and 85 for *Homo sapiens*, 73 and 87 for *Oxyuranus scutellatus* and 79 and 88 for *Caenorhabditis elegans*. All of the seven conserved amino acid blocks defining the HSP90 protein family signature GVVDS (E/D) DLPLN (I/V) SRE (Figure 1, underlined black-box) and the consensus sequence MEEVD (Figure 1, underlined white-box) at the C-terminus were found in the Bx-HSP90 sequence.

**Figure 2:**
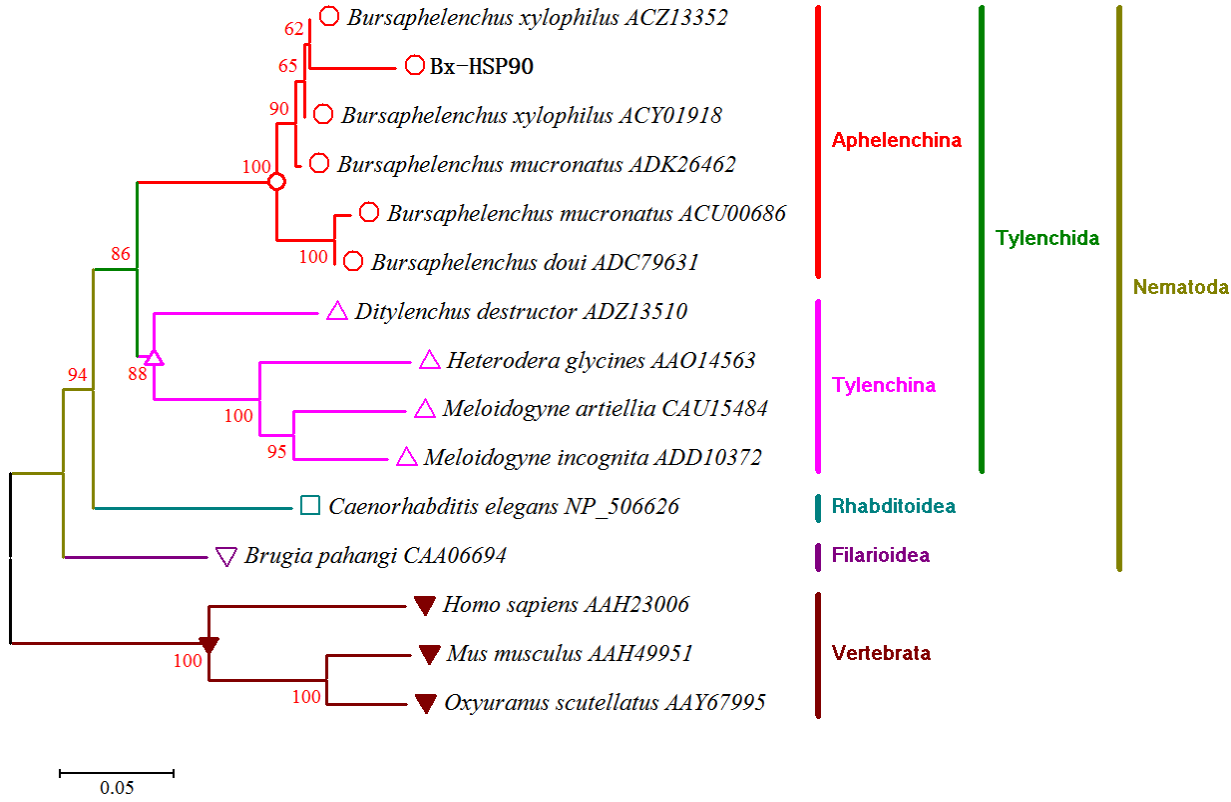
Phylogenetic tree showing the relationship between Bx-HSP90 and the HSP90s from other parasitic or free-living nematodes and from the Vertebrata outgroup. One thousand bootstrap replicates were performed, and the node labels represent the percent bootstrap support.

A phylogenetic tree was constructed based on the HSP90 sequences of various organisms (Figure 2) using the MEGA 5.05 program, and the resulting tree showed five major clusters. The Bx-HSP90 sequence was closely clustered with the homologous sequences of other *Bursaphelenchus* species (*B. xylophilus*, *B. mucronatus* and *B. doui*).

We searched the *B. xylophilus* genome (CADV01000001-CADV01010432) [12] with the Bx-HSP90 protein sequence using the TBLASTN algorithm. Using an expectation value of 1e-006, this TBLASTN homology search identified seven alignments located in contigs CADV01010217.1 (Score: 280, E Value: 3 × 10^−75^), CADV01010388.1 (Score: 75, E Value: 1 × 10^−13^), CADV01008131.1 (Score: 44, E Value: 1 × 10^−8^), CADV01007052.1 (Score: 39, E Value: 6 × 10^−8^), CADV01003002.1 (Score: 39, E Value: 8 × 10^−8^), CADV01005604.1 (Score: 38, E Value: 1 × 10^−7^) and CADV01005840.1 (Score: 54, E Value: 4 × 10^−7^). Except for CADV01010217.1, which is the *Bx-HSP90* contig, the other alignments are unlikely to be related to homologous sequences putatively coding for *HSP90*s. *HSP90* is a single-copy gene in the *C. elegans* and *B. pahangi* genomes [13,14], and the results presented here indicate that *Bx-HSP90* might also be a single-copy gene in the *B. xylophilus* genome.

### 2.2. *In Situ* Hybridization

**Figure 3:**
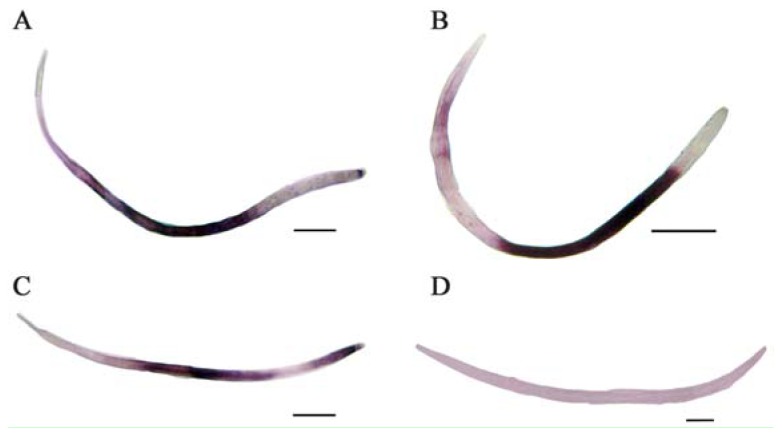
Localization of *Bx-HSP90* mRNA by *in situ* hybridization using a digoxygenin-labeled *Bx-HSP90* antisense or sense probe. (**A**) Antisense probe in a 25 °C heat-shocked nematode. (**B**) Antisense probe in a 30 °C heat-shocked nematode. (**C**) Antisense probe in a 35 °C heat-shocked nematode. (**D**) Control sense probe in a 30 °C heat-shocked nematode. The scale bars represent 100 μm.

*In situ* hybridization was used to determine the tissue specificity of *Bx-HSP90* transcription. In heat-shocked nematodes, the *Bx-HSP90* anti-sense probe showed localized hybridization in an area of approximately 350 μm in length and 20 μm in width located over approximately 35% of the body length in the middle of the nematode (Figure 3A–C). The hybridization signals were detected at 25, 30 and 35 °C (Figure 3A–C, respectively). No equivalent hybridization was observed using the control sense probe (Figure 3D). The data showed that the *Bx-HSP90* gene was constitutively expressed in response to all of the tested temperatures.

### 2.3. Analysis of Transcript Abundance

The nematode survival rate was determined after 24 h of incubation at 25, 30 and 35 °C using the loss of spontaneous and touch-provoked movement to indicate death. We found that 100, 99 and 46% of the nematodes were alive at 25, 30 and 35 °C, respectively. Both 30 and 35 °C constitute high-temperature stress for *B. xylophilus*, although only a slight decrease in survival occurred at 30 °C within the 24-h period.

**Figure 4:**
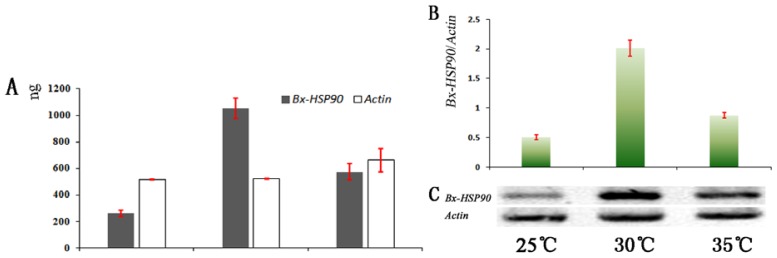
Analysis of the transcript abundance for *Bx-HSP90* after the exposure of *B. xylophilus* to 25, 30 and 35 °C for 24 h. (**A**) Quantitative evaluation of *Bx-HSP90* and *Actin* expression using the PCR product signals measured by BandScan 5.0. (**B**) *Bx-HSP90*/*Actin* ratios. (**C**) *Bx-HSP90* mRNA transcription relative to *Actin* mRNA analyzed by RT-PCR.

To validate a relationship between nematode survival and the *Bx-HSP90* transcript levels, RT-PCR analyses were performed using the total RNA from *B. xylophilus* incubated at 25, 30 and 35 °C for 24 h. Three biological replicates were conducted in this experiment, and the signals of the PCR product were measured using BandScan 5.0. The *Bx-HSP90* transcript signal was detected in all of the temperature treatments, with the highest level detected at 30 °C (Figure 4). Compared with the level at 25 °C, the *Bx-HSP90* transcript level was significantly increased at 30 °C but only slightly increased at 35 °C. After 24 h, *Bx-HSP90* transcription in the heat-shocked nematodes displayed a temperature-dependent pattern (Figure 4C *Bx-HSP90*): The level of *Bx-HSP90* mRNA increased as the temperature increased from 25 to 30 °C and decreased as the temperature increased from 30 to 35 °C. The transcription of the Actin gene was used as a control, and the amount of *Actin* transcript was similar in all of the treatments (Figure 4C *Actin*). This stable transcription of *Actin* suggested that the *Bx-HSP90* RNA was qualified. The transcripts of the *Bx-HSP90* gene appear to be significantly up-regulated in response to heat shock. Together with the survival data, our observations suggest that the production of the highest level of *Bx-HSP90* at 30 °C may be adaptive in maintaining a native protein structure under high-temperature stress. At 35 °C, we found that the *Bx-HSP90* transcript level also increased compared with the level produced at 25 °C. Contrary to our expectation, however, nematode survival did not increase with this increase in transcript but sharply decreased. These results suggest that *Bx-HSP90* was only weakly active at 35 °C such that it could not fulfill a thermoregulatory function. Thus, *Bx-HSP90* does play a role in thermoregulation but functions maximally within a restricted temperature range.

### 2.4. RNAi of *Bx-HSP90*

**Figure 5:**
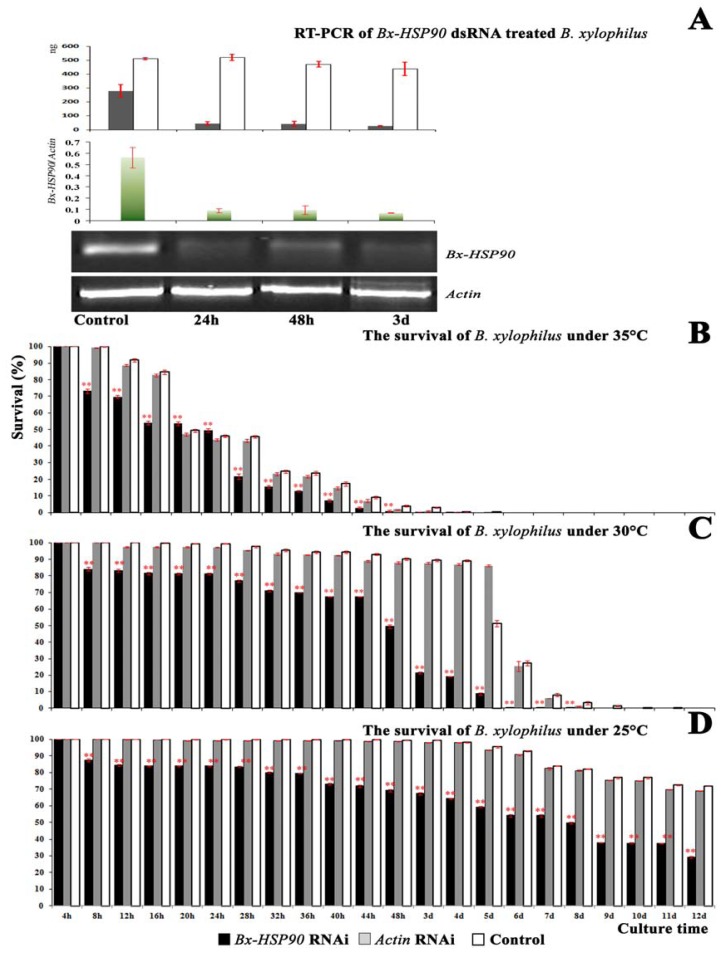
Transcript abundance analysis and survival of *Bx-HSP90* RNAi-treated *B. xylophilus*. (**A**) RT-PCR using *Bx-HSP90* RNAi-treated *B. xylophilus* RNA as the template to determine the extent of *Bx-HSP90* RNAi. From top to bottom, the signals detected for quantitative evaluation of the RT-PCR products, the *Bx-HSP90*/*Actin* ratios of *B. xylophilus* and photographs of the RT-PCR products. (**B**), (**C**) and (**D**) Survival of the RNAi-treated and control *B. xylophilus* exposed to temperatures of 35, 30 and 25 °C. At each temperature, the survival of the *Bx-HSP90* RNAi-treated nematodes was significantly different from that of the control (** *p* < 0.01, *n* = 5).

There have been reports suggesting that *HSP90* is essential in the filarial nematode, *B. pahangi*, and in the free-living nematode, *C. elegans* [14]. Because the knockout of *HSP90* is lethal in eukaryotes, we used regular temperatures and high-temperature stress environments to examine whether *HSP90* had any effect on the mortality of *B. xylophilus*. The RNA interference of *Bx-HSP90* was performed for mixed-stage *B. xylophilus* using the method developed by Urwin *et al*. (2002) to allow the octopamine-stimulated uptake of double-stranded RNA (dsRNA). The patterns of fluorescein isothiocyanate (FITC) uptake observed here for the nematodes incubated in octopamine and FITC were similar to those described by Urwin *et al*. (2002) [15]. RT-PCR experiments were performed to determine the extent of *Bx-HSP90* RNAi in *B. xylophilus*. Although a significant *Bx-HSP90* silencing was found after soaking in the relevant dsRNA (Figure 5A *Bx-HSP90* at 24 h, 48 h and 3 day), the *Bx-HSP90* dsRNA had no effect on the transcription of *Actin* (Figure 5A *Actin*). From these data, we conclude that the RNAi by soaking was potent and specific for *B. xylophilus*.

The survival of the nematodes subjected to *Bx-HSP90* RNAi treatment decreased to 50% after incubation for 8 days at 25 °C (Figure 5D). A similar reduction was observed after 48 h at 30 °C and after 24 h at 35 °C (Figure 5B,C). The RNAi treatment produced a 25% reduction in survival at 30 °C after 44 h: more than 90% of the RNAi-treated nematodes were dead upon the exposure to 30 °C for 5 days compared with 7 days for the controls. A *t*-test for paired samples showed that the survival of the *Bx-HSP90* RNAi-treated nematodes differed significantly from that of the control nematodes, and a bivariate correlation analysis indicated a significant correlation between survival and the temperature (*p* < 0.01, *Bx-HSP90* RNAi *vs* controls). The *Actin* RNAi negatively affected the survival of the nematodes, but there was no correlation between the survival and the temperature treatment. These data suggest that the HSP90 gene might function in thermoregulation for specific durations within a defined range of high temperatures. Judging from the *Bx-HSP90* RNAi results, combined with the RT-PCR results, *Bx-HSP90* apparently functions strongly for at least 5 days at 30 °C but poorly at 35 °C.

**Figure 6:**
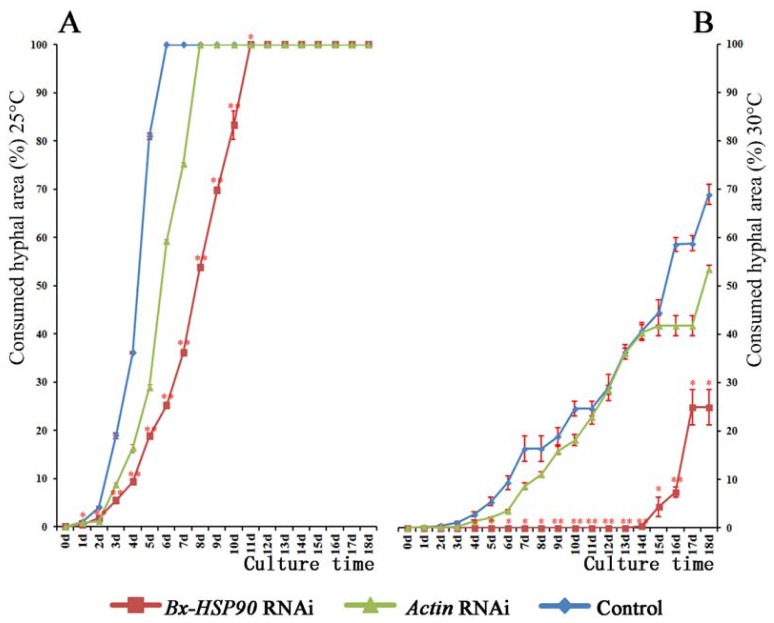
Area of *B. cinerea* hyphae consumed by RNAi-treated and control *B. xylophilus*. The nematodes were cultured at 25 °C (**A**) or 30 °C (**B**) (* *p* < 0.05, **p < 0.01, *n* = 3).

To investigate the relationship between *Bx-HSP90* and the nematode population growth, we measured the hyphal mat consumed by control, *Bx-HSP90* RNAi-treated and *Actin* RNAi-treated *B. xylophilus*, which were cultured on fungi (*B. cinerea*) at 25, 30 and 35 °C. The measurement of the area of the hyphal mat that was consumed permitted a quantitative estimation of the population growth of the nematode. At the 6th day at 25 °C, the *Bx-HSP90* RNAi-treated *B. xylophilus* consumed only 25% of the hyphae, whereas the *Actin* RNAi-treated and the control nematodes consumed 58 and 100%, respectively. The *Bx-HSP90* RNAi-treated *B. xylophilus* required 11 days to consume all of the hyphae at 25 °C, and the *Actin* RNAi-treated *B. xylophilus* required 8 days. These results demonstrate that both the *Bx-HSP90* and *Actin* RNAi-treated *B. xylophilus* grew normally at 25 °C but that their population growth rates were lower than that of the control. At the 14th day, no hyphae were consumed in the culture dishes containing the *Bx-HSP90* RNAi-treated nematodes at 30 °C. The population growth rates of both the *Bx-HSP90* RNAi-treated and *Actin* RNAi-treated *B. xylophilus* were slower at 30 than at 25 °C, with the rate being slower for the *Bx-HSP90* RNAi-treated group. After 14 days, the populations of the *Bx-HSP90* RNAi-treated *B. xylophilus* had not grow at 30 °C, whereas the control populations grew at that temperature beginning at the 2nd day (Figure 6B). Regardless of the treatment, no consumption of hyphae occurred at 35 °C during the 18 days observation period.

The temperature regulation of gene expression is critical for the survival and success of organisms living in thermally variable environments. Heat shock proteins function during cell stress as molecular chaperones, interacting with diverse protein substrates to assist in repairing damaged proteins by refolding or degrading them, thereby restoring protein homeostasis and promoting cell survival [16]. Our results have implications for the roles that Bx-HSP90 may play in the environmental adaptation processes of *B. xylophilus*; for example, the protein may help to buffer the stress of high temperatures (within a certain high-temperature range, which includes 30 °C). However, further studies are necessary to clarify this point and to elucidate the possible involvement of other stress proteins in mitigating a vulnerability to the multitude of environmental stresses affecting development.

Using RNAi, we showed that *Bx-HSP90* is essential for the survival of the nematode *B. xylophilus*. HSP90 can be considered important for nematodes because silencing it reduces their survival at all temperatures. These results demonstrate that the inhibition of HSP90 using such agents as geldanamycin may represent a novel approach for the treatment of pine wilt disease. HSP90 or some of its client proteins of mosquito and mammalian parasites, such as the filarial nematode, *B. pahangi*, have been proposed as drug targets [15]. Because HSP90 is associated with diverse tissues and functions within nematodes, there is the prospect of affecting a range of processes using the control agents that target this protein. The inhibition of HSPs leads to reduced growth and fecundity of plant parasitic nematodes [17]. If the survival of nematodes can be reduced at warmer temperatures, even by a small percentage, this approach may be effectively used against *B. xylophilus* in warmer climates.

## 3. Experimental Section

### 3.1. Nematode Culture and Heat Shock

*B. xylophilus* specimens were isolated from wilted *Pinus massoniana* in the Guangdong Province of China using the Baermann funnel technique. The nematodes were cultured on the *Botrytis cinerea* for 5 days at 25 °C and then divided into two groups. One group was heat shocked at 30 °C for 24 h to induce the expression of thermotolerance-related genes, and a second group was cultured at 25 °C as a control.

### 3.2. Construction of the Subtracted cDNA Libraries and Sequence Analyses

For the RNA extractions, the shocked nematodes and controls were separately frozen in a mortar with liquid nitrogen and powdered using a pestle. For each group, the total RNA was extracted from 100 μg of mixed nematode stages (a mixture of adult and juvenile nematodes in a male to female to juvenile ratio of approx. 1:1:2) using the TRIzol reagent (Invitrogen, USA, cat. No. 15596-026). The RNA was then purified using DNase I (RNase-free) (TaKaRa, Japan, cat. No. D2270A) treatment. Double-stranded cDNAs were generated using a SMART (switching mechanism at the 5′ end of the RNA transcript) kit according to instructions of the manufacturer (BD Clontech, USA, cat. No. 634902).

The PCR-select cDNA subtraction was performed using the BD PCR-Select cDNA Subtraction Kit (BD Clontech, USA, cat. No. PT1117-1) according to established protocols [18]. The cDNA from the heat-shocked nematodes was used as the tester, and the cDNA from the untreated controls was used as the driver. The cDNAs were digested with *Rsa* I and then ligated to different adapters. Two rounds of hybridization and PCR amplification were processed to normalize and enrich the differentially expressed cDNAs. The products of the secondary PCR from the forward subtraction were directly inserted into the pGEM-T vector and transformed into *Escherichia coli* JM109 competent cells (Promega, USA, cat. No. A3610). All of the recombinant clones were included to establish the subtracted cDNA library. Random sequencing of the library using the T7 primer yielded 89 successful sequencing reactions.

The BLAST analysis of all 89 of the sequences revealed that one sequence of 893 bp (sequence No. *B40*) was homologous to the HSP90 gene of other species. This sequence was selected for further cloning of the full-length cDNA of the HSP90 gene from *B. xylophilus*. None of the other sequences showed a direct relationship with *HSP90* (data not show).

### 3.3. Cloning of *Bx-HSP90*

Most nematode messenger RNAs (mRNAs) have a short sequence at their 5′ end that is not contiguously encoded in the genome. This leader sequence is trans-spliced to the 5′ end of primary transcripts during the maturation of the pre-mRNA. This spliced leader (SL) provides a 5′ anchor, which both simplifies the process of second-strand cDNA synthesis and allows the isolation of cDNA molecules that are guaranteed to be full length [19]. The SL primer PCR used a spliced leader sequence 5′ (forward) primer, SL1 [20], and the specific primer SLHSP90-A2L was used to amplify the entire transcriptome. To obtain a complete sequence of this gene, 3′ RACE (rapid amplification of cDNA ends) techniques were performed using the specific primer SLHSP90-S1L with the SMART RACE cDNA Amplification Kit (Clontech, USA, cat. No. K1811-1) according to the manufacturer’s instructions.

The sequences obtained from the spliced leader primer PCR and the 3′-RACE were joined with that of *B40* to generate a full cDNA sequence, which was named *Bx-HSP90*. The full-length cDNA obtained was PCR amplified using the forward primer EHSP90S2L and the reverse primer EHSP90A1L, which contained start and stop codons at the 5′ and 3′ ends, respectively.

### 3.4. Homology Analysis

Searches for nucleotide and amino acid sequence similarities were conducted using the BLAST programs at the National Center for Biotechnology Information (NCBI) [21]. Multiple alignments of HSP90 were performed using the ClustalW multiple alignment program [22]. The similarity percentages of the full-length amino acid sequences between HSP90 and other HSP90s were calculated using the Identity and Similarity Analysis program [23]). The signal peptide was predicted with SignalP 3.0 [24]. To find the conserved HSP90 domains, the amino acid sequences of the HSP90 proteins that have been analyzed (*H. glycines*, *B. pahangi*, *M. musculus*, *H. sapiens*, *O. scutellatus* and *C. elegans*) were aligned with Bx-HSP90 using the BioEdi program. Those aligned sequences and other homologous sequences from plant nematode species (*Ditylenchus destructor*, ADZ13510; *Meloidogyne incognita*, ADD10372; *M. artiellia*, CAU15484), including several *Bursaphelenchus* species (*B. xylophilus*, ACZ13352 and ACY01918; *B. mucronatus*, ACU00686 and ADK26462; *B. doui*, ADC79631) were all retrieved from NCBI and then used to generate a phylogenetic tree using the maximum-likelihood method with MEGA 5.05.

Homologous sequences were found using Bx-HSP90 as a query sequence in a TBLASTN search of the *B. xylophilus* complete genome sequence databases (the *B. xylophilus* whole-genome shotgun project consists of sequences CADV01000001-CADV01010432) [12].

### 3.5. *In Situ* Hybridization

The nematodes were heat shocked at 25, 30 or 35 °C for 24 h to induce *Bx-HSP90* expression. *In situ* hybridization was performed on the heat-shocked *B. xylophilus* essentially as described in De Boer *et al*. [25]. A digoxigenin-labeled RNA probe was synthesized using the cDNA sequence following the general protocol of the Roche DIG RNA Labeling Kit (SP6/T7) (Roche, Germany, cat. No. 1175025). The specificity of the probe was verified by Northern blotting. The hybridization signals were detected by incubation with the DIG High Prime DNA Labeling and Detection Starter Kit I (Roche, Germany, cat. No. 11745832910) and photographed under a microscope (Olympus Bx51, Japan). The backgrounds of the images were cropped using Adobe Photoshop CS2.

### 3.6. Analysis of Transcript Abundance

The expression of the *Bx-HSP90* transcript in the heat-shocked *B. xylophilus* was measured using RT-PCR. The nematodes were cultured at 25, 30 and 35 °C for 24 h; three biological replicates were used in this experiment. The fresh living nematodes for the RNA extraction were all collected at the same temperature using the Baermann funnel technique. Two *Bx-HSP90* gene-specific primers, HSP90rtR1L and HSP90rtF1L (Table 1), were used to amplify a 405-bp product. A constitutively expressed gene, *Actin*, was used as an internal control to verify the quantitative RT-PCR reaction using primers (RsACT_F and RsACT_R) (Table 1) [26]. PCR-grade water replaced the cDNA template in the negative control. The RT-PCR was performed for 28 cycles, and the signals of the RT-PCR products were measured using BandScan 5.0. The 150-ng (750 bp) band of the DL 2,000 DNA marker (TaKaRa, Japan, cat. no. D500A) was used as a quantitative standard band. The survival of the heat-shocked nematodes was recorded to measure the relationship between *Bx-HSP90* and mortality.

### 3.7. RNAi of *Bx-HSP90*

The dependence of the observed thermotolerance on the *Bx-HSP90* gene was estimated by the RNA interference of the function of the gene. The RNAi was performed using mixed stages of the nematode (a mixture of adult and juvenile nematodes in a male-to-female-to-juvenile ratio of approx. 1:1:2), as outlined in Urwin, *et al*. [13]. Double-stranded RNA corresponding to *Bx-HSP90* was prepared using the MAXIscript T7/T3 RNA Synthesis Kit (Ambion, Japan, cat. no. AM1324M). The treated nematodes were soaked in M9 buffer with 10 mM octopamine and dsRNA (5 mg/mL) corresponding to the *Bx-HSP90* sequence. The uptake of the soaking solution was monitored by a final nematode treatment of M9 buffer containing 10 mM octopamine and 1 mg/mL FITC. The control nematodes were soaked in M9 buffer only, M9 buffer with 10 mM octopamine or *Actin* dsRNA (5 mg/mL). After 12 h of soaking with intermittent agitation at 25 °C, the nematodes were washed copiously with sterile water to remove the external dsRNA and then observed fluoroscopically to detect the uptake of FITC.

The RNAi-treated nematodes were divided into three groups: The first group was used to determine the extent of *Bx-HSP90* RNAi; the second was used to assess survival under 25, 30 and 35 °C heat shock; and the third was used to assess the population growth under 25, 30 and 35 °C heat shock.

RNA was extracted from the control nematodes and the RNAi-treated nematodes after 24 h, 48 h and 3 days. RT-PCR was then performed using the following primers: HSP90rtR1L, HSP90rtF1L, RsACT_F and RsACT_R (Table 1).

The survival of the RNAi-treated nematodes and controls was assessed during their culture at 25, 30 and 35 °C (*n* = 500 for each temperature) and was recorded every 4 h for 2 days and then daily for 12 days. Each treatment was repeated four times. A *t*-test for paired samples and a bivariate correlation analysis (SPSS 13.0) were used to determine the extent to which RNAi influenced the number of nematodes surviving the exposure to the experimental temperatures.

Samples of 100 RNAi-treated nematodes (a mixture of adults and juveniles in a male-to-female-to-juvenile ratio of approx. 1:1:2) or controls were cultured on *B. cinerea* at 25, 30 and 35 °C to measure the relationship between the function of *Bx-HSP90* and mortality. Each treatment was cultured in 10 dishes, and the process repeated twice. Images of the culture dishes were obtained using a digital camera (Nikon, Japan) every day for 18 days. The area of the hyphal mat that was consumed in each dish was measured using ImageJ 1.45 m [27,28]. Because the area of the *B. cinerea* hyphal mat that is consumed is proportional to the number of *B. xylophilus* feeding on the mat, this technique quantitatively estimated the population growth of the nematodes in each dish.

## 4. Conclusions

In summary, we cloned a thermotolerance-related gene, *Bx-HSP90*, from *B. xylophilus* and investigated its function. Our study shows that *Bx-HSP90* plays an important role in *B. xylophilus*. The heat shock and knockdown of *Bx-HSP90* both hindered the population growth of *B. xylophilus*. The knockdown of this gene was lethal at 30 °C and 35 °C, whereas the expression of *Bx-HSP90* increased the survival and population growth rate of *B. xylophilus* under higher temperature conditions (30 °C). These increases were statistically significant at 30 °C, but the expression of *Bx-HSP90* had little effect at 35 °C. These data suggest that the up-regulation of *Bx-HSP90* expression can increase the thermotolerance of *B. xylophilus* within a certain temperature range and that agents able to disable *Bx-HSP90* function could prove useful to control *B. xylophilus* in the future.

## Figures and Tables

**Table 1:** Primers used in this study.

Primer Name	Primer Sequence	Reference
SL1	5′-GGTTTAATTACCCAAGTTTGAG-3′	Blaxter *et al*. [20]
SLHSP90-A2L	5′-GTCAAGAGGGCGGTCTCGA-3′	This study
SLHSP90-S1L	5′-CACCTCCGGAGACGAGACC-3′	This study
EHSP90S2L	5′-ACTTCATCATGGAGAACTGCG-3′	This study
EHSP90A1L	5′-ATCATTAGGAGGACAACAGA-3′	This study
HSP90rtR1L	5′-GCGATGAACTGATGCCCGACTAC-3′	This study
HSP90rtF1L	5′-CGAAGGCAGAGTTGGCGACG-3′	This study
RsACT_F	5′-GAAAGAGGGCCGGAAGAG-3′	Joachim *et al*. [26]
RsACT_R	5′-AGATCGTCCGCGACATAAAG-3′	Joachim *et al*. [26]
